# Proteomic and phosphoproteomic characterization of cardiovascular tissues after long term exposure to simulated space radiation

**DOI:** 10.3389/fphys.2024.1248276

**Published:** 2024-04-18

**Authors:** Yared H. Kidane, Franklin H. Lee, Matthew F. Smith, Chunbo Wang, Jacqueline Barbera Mirza, Saachi Sharma, Alejandro A. Lobo, Krish C. Dewan, Jengwei Chen, Thomas E. Diaz, Michelle Mendiola Pla, Matthew W. Foster, Dawn E. Bowles

**Affiliations:** ^1^ Center for Pediatric Bone Biology and Translational Research, Scottish Rite for Children, Dallas, TX, United States; ^2^ Department of Surgery, Duke University Medical Center, Durham, NC, United States; ^3^ Dr. Kiran C. Patel College of Allopathic Medicine, Nova Southeastern University, Fort Lauderdale, FL, United States; ^4^ Stanton College Preparatory School, Jacksonville, FL, United States; ^5^ Department of Surgery, National Taiwan University Hospital, Taipei, Taiwan; ^6^ Eshelman School of Pharmacy, Chapel Hill, NC, United States; ^7^ Duke Proteomics and Metabolomics Core Facility, Duke University Medical Center, Durham, NC, United States

**Keywords:** galactic cosmic ray, cardiovascular degeneration, space radiation, proteomics, mass spectrometry, ionizing radiation, cardiovascular disease, phosphoproteomics

## Abstract

**Introduction:** It may take decades to develop cardiovascular dysfunction following exposure to high doses of ionizing radiation from medical therapy or from nuclear accidents. Since astronauts may be exposed continually to a complex space radiation environment unlike that experienced on Earth, it is unresolved whether there is a risk to cardiovascular health during long-term space exploration missions. Previously, we have described that mice exposed to a single dose of simplified Galactic Cosmic Ray (GCR_5-ion_) develop cardiovascular dysfunction by 12 months post-radiation.

**Methods:** To investigate the biological basis of this dysfunction, here we performed a quantitative mass spectrometry-based proteomics analysis of heart tissue (proteome and phosphoproteome) and plasma (proteome only) from these mice at 8 months post-radiation.

**Results:** Differentially expressed proteins (DEPs) for irradiated versus sham irradiated samples (fold-change ≥1.2 and an adjusted *p*-value of ≤0.05) were identified for each proteomics data set. For the heart proteome, there were 87 significant DEPs (11 upregulated and 76 downregulated); for the heart phosphoproteome, there were 60 significant differentially phosphorylated peptides (17 upregulated and 43 downregulated); and for the plasma proteome, there was only one upregulated protein. A Gene Set Enrichment Analysis (GSEA) technique that assesses canonical pathways from BIOCARTA, KEGG, PID, REACTOME, and WikiPathways revealed significant perturbation in pathways in each data set. For the heart proteome, 166 pathways were significantly altered (36 upregulated and 130 downregulated); for the plasma proteome, there were 73 pathways significantly altered (25 upregulated and 48 downregulated); and for the phosphoproteome, there were 223 pathways significantly affected at 0.1 adjusted *p*-value cutoff. Pathways related to inflammation were the most highly perturbed in the heart and plasma. In line with sustained inflammation, neutrophil extracellular traps (NETs) were demonstrated to be increased in GCR_5-ion_ irradiated hearts at 12-month post irradiation. NETs play a fundamental role in combating bacterial pathogens, modulating inflammatory responses, inflicting damage on healthy tissues, and escalating vascular thrombosis.

**Discussion:** These findings suggest that a single exposure to GCR_5-ion_ results in long-lasting changes in the proteome and that these proteomic changes can potentiate acute and chronic health issues for astronauts, such as what we have previously described with late cardiac dysfunction in these mice.

## Introduction

We recently reported on long term functional changes to the heart following a single dose of GCR_5-ion_ (Bishawi et al., 2022). Physiologically, this dysfunction was manifested as changes in arterial elastance, and histologically, there was evidence of elastin fragmentation in the aorta. Of the protein markers we evaluated (Fibulin-4, Fibulin-5, TGF-beta1, and EGFR), EGFR was the only marker that showed a statistically significant decrease in hearts from GCR-irradiated animals compared to age-matched sham irradiated controls (Bishawi et al., 2022).

Other investigations have also examined the levels of individual cardiac protein markers in studies where rodents or rabbits were exposed to components of GCR (reviewed in Davis et al., 2021; Seawright et al., 2019; Amino et al., 2010; Soucy et al., 2011). These studies have indicated that a number of proteins and pathways and biological functions are permanently affected in the heart from a single exposure of space relevant radiation. Seawright et al. (Seawright et al., 2019) noted an increase in a 75 kDa peptide of collagen type III in the left ventricle of rats exposed to protons (250 MeV) and ^16^O (600 MeV/n and 1 GeV/n), suggesting that these components of GCR caused remodeling of existing collagens in the heart. Also noted in this study were increases in the amounts of CD2, CD68, and CD45 proteins at 2 weeks, 3 months, and 9 months after exposure to protons or ^16^O alone, suggesting immune cell infiltration. In a rabbit model, hearts exposed to 15 Gy of targeted heavy ion irradiation (THIR) exhibited a significant upregulation of connexin 43 (Cx43) protein and mRNA in the ventricular myocardium by immunohistochemistry, Western blotting, and real-time PCR from 2 weeks out to 1 year after a single exposure (Amino et al., 2010). Cx43, the major connexin in cardiac gap junction, plays an essential role in the synchronized contraction of the heart and perturbations in Cx43 levels could profoundly influence cardiac function.

Soucy et al. examined xanthine oxidase (XO) activity in the aortas of rats exposed to whole-body exposure of iron ion radiation (^56^Fe-ion) at doses of 0, 0.5, or 1 Gy (Soucy et al., 2011). *In vivo* aortic stiffness and *ex vivo* aortic tension responses were measured 6 and 8 months after exposure as indicators of chronic vascular injury. Rats exposed to 1 Gy ^56^Fe-ion demonstrated significantly increased aortic stiffness, as measured by pulse wave velocity. XO activity was significantly elevated in rat aorta 4 months after whole-body irradiation.

Recently, Azimzadeh et al. published a comprehensive review of the available omics data (proteomics, transcriptomics, metabolomics, epigenomics, and multi-omics) of radiation-induced cardiovascular disease focusing on gamma and X-ray exposure (Azimzadeh et al., 2022). Across 27 published proteomics data sets, mitochondrial proteins were the most sensitive protein cluster affected in irradiated hearts across different types and doses of radiation, with many of these mitochondrial proteins being significantly downregulated. In addition, long term downregulation of many anti-oxidant defense proteins was observed in many publication examining mouse models as well as validated in studies of heart autopsies obtained from workers in the nuclear industry.

While recently Laiakis et al (2022) have utilized label-free mass spectrometry untargeted quantitative proteomic profiling to characterize the proteome of the prefrontal cortex in rats exposed to ^28^Si ions, there have been no corresponding studies undertaken to understand the global proteomic changes occurring in the heart following exposure to components of space radiation. As the proteome can provide a better understanding of the structure and the function of the organism than genomics, we have performed a quantitative mass spectrometry-based proteomics analysis of heart tissue (proteome and phosphoproteome) and plasma (proteome only) from mice exposed to GCR_5-ion_. Integration of these three omics data sets has enabled a more comprehensive understanding of the systemic impact of GCR_5-ion_ and suggests that sustained inflammatory response with neutrophil extracellular trap formation is one of the major pathways driving the biological responses to space radiation in the heart.

## Methods

**FIGURE 1:**
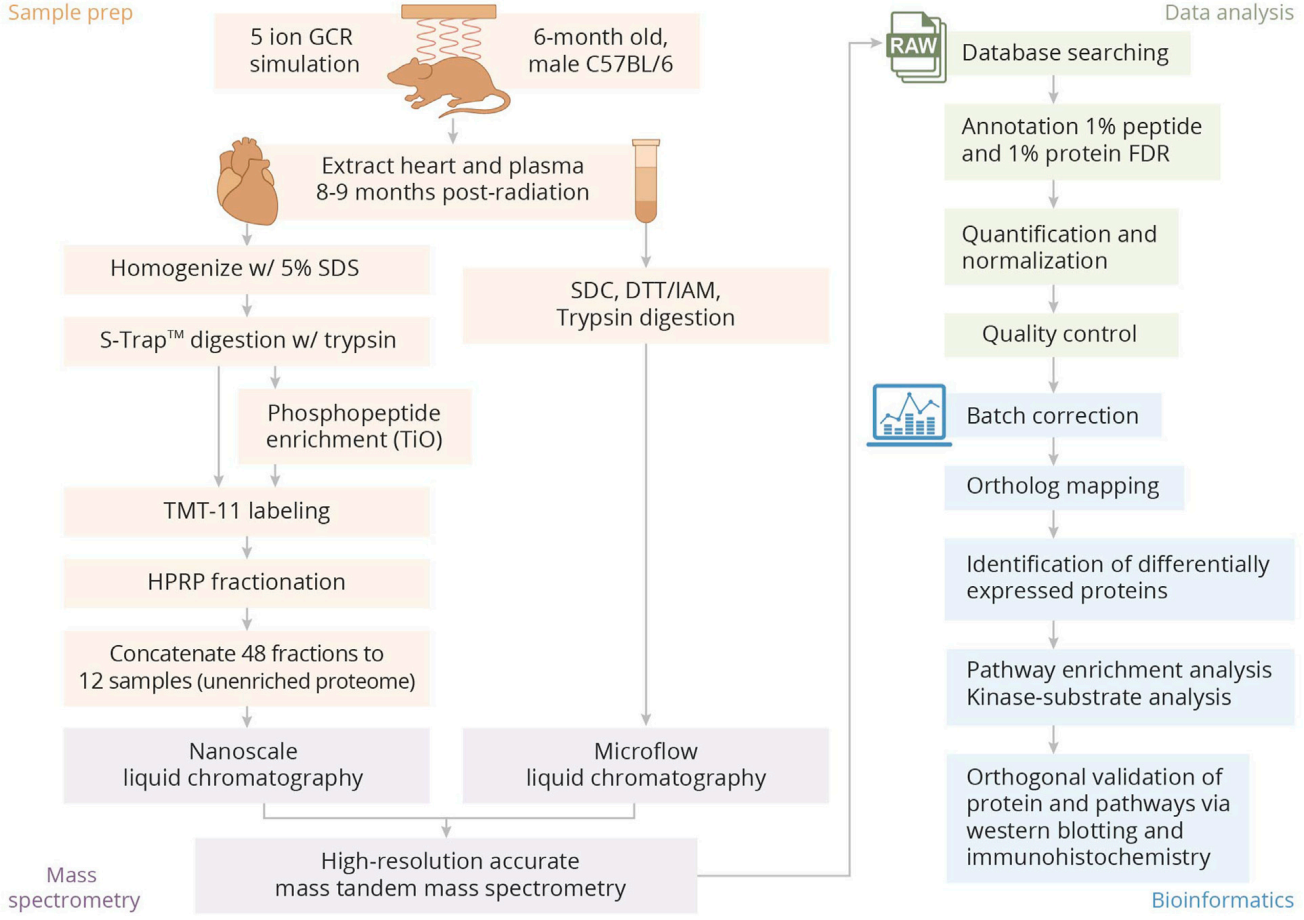
Overview of sample preparation, processing, and bioinformatics analysis pipeline.

The overall experimental design is provided in Figure 1.

### Experimental animals

All animal experiments were approved by the Duke University and Brookhaven National Laboratory (BNL) Institutional Animal Care and Use Committees (IACUCs). Approximately 24-week old male C57BL/6 mice (Jackson Labs) were exposed to 150 cGy GCR_5-ion_ (H 1,000 MeV/n, ^28^Si 600 MeV/n, ^4^He 250 MeV/n, ^16^O 350 MeV/n, ^56^Fe 600 MeV/n, and H 250 MeV/n) using the beam line at the NASA Space Radiation Laboratory (NSRL) at BNL (Simonsen et al., 2020). Sham irradiated control animals were placed in equitable holders over a schedule which mimicked the experimental groups but were not irradiated. Post-radiation, animals were allowed at least 2 days to recover, and then were returned to Duke University animal facilities. Hearts and plasma for proteomics were obtained from mice at 8 months post radiation (n = 6 for hearts and n = 10 for plasma). An equal number of hearts and plasma samples was obtained from age-matched sham irradiated control animals. Hearts for other evaluations such as Western blot or immunofluorescence studies were obtained from mice at 12 months post radiation and equivalent age-matched sham irradiated control animals were used as comparators.

#### Heart tissue (phospho)proteomics

Frozen tissues were weighed and lysed by tissue tearor in 1.5 mL of 5% sodium docecyl sulfate (SDS)/50 mM triethylammonium bicarbonate, pH 8.5 (TEAB) followed by bicinchoninic acid (BCA) assay. The amount of 250 μg of each sample was normalized to 50 μL with SDS/TEAB buffer followed by the addition of bovine casein (300 fmol and 150 fmol per µg added to irradiated and sham irradiated control samples, respectively) and digestion using an S-trap mini device, followed by Tandem Mass Tag (TMT) labeling (with lot# TK2735U and TL272831), high pH-reversed phase (HPRP) fractionation, and phosphopeptide enrichment as previously described (Walejko et al., 2021).

One-dimensional liquid chromatography tandem mass spectrometry (1D-LC-MS/MS) was performed on ∼0.5 μg each of the unenriched fractions, and 2.5 μL of the phosphopeptide-enriched fractions were analyzed using a nanoACQUITY UPLC system (Waters) coupled to a Fusion Lumos high resolution accurate mass tandem mass spectrometer (Thermo) in a time-dependent (3 s) data-dependent acquisition (DDA) mode with a 120,000 resolution (@ m/z 200) full MS scan from m/z 375 to 1,600 with a target automatic gain control (AGC) value of 2e5 ions and 50 ms maximum injection time (IT) and internal calibration (eIC) enabled. Peptides were selected for MS/MS using monoisotopic precursor selection (MIPS) and a dynamic exclusion of 45 s. Peptides with charge state 2 (scan priority 1) were selected for MS/MS using an isolation width of 1.2 and higher energy collision-induced dissociation (HCD) with a stepped normalized collision energy of 36.1, 38, and 39.9 with detection in the orbitrap using a resolution of 50,000, an AGC target of 1e5, and max IT of 105 ms. Peptides with charge states 3–5 (scan priority 2) were selected for MS/MS with an isolation window of 0.7 m/z.

Phosphoproteome data were analyzed using Thermo Proteome Discoverer 2.3. Re-calibrated.mgf Calibrated .mgf files were created the Spectrum Files RC node and database searching was performed against the Uniprot database with *Mus musculus* taxonomy (downloaded on 04/22/19; 20,204 entries) appended with bovine casein isoform protein sequences. Searches used Mascot v 2.5 with 5 ppm precursor and 0.02 product ion tolerances, trypsin specificity with up to two missed cleavages, fixed carbamidomethylation (Cys) and TMT10 (Lys and peptide N-terminal), and variable phosphorylation (Ser, Thr, and Tyr). Percolator and imp-ptmRS nodes were used for FDR determination and site localization. The processing node of the reporter ions quantifier used a 10 ppm integration tolerance, and the consensus node used correction for isotope impurity, a co-isolation threshold of 50%, and S/N threshold of 5. Normalization to total peptide amount and imputation using low abundance resampling were applied.

Proteome data was analyzed in Proteome Discoverer 2.4 using the MS-Fragger (Chang et al., 2023) and Percolator nodes with default parameters. The mouse database was appended with additional contaminant sequences including human keratins. Imputation used low abundance resampling, and protein quantities were calculated from summed peptide abundances. Normalization used sample loading and trimmed mean in R as previously described (Wakeham et al., 2019).

#### Plasma proteomics

Plasma proteomics utilized sodium deoxycholate (SDC)-assisted in solution digestion followed by microflow liquid chromatography coupled to tandem mass spectrometry using variable window data-independent acquisition (DIA) as previously described (Kamyszek et al., 2020) with the following modifications. Briefly, 15 μL of plasma was adjusted to 5% SDC in 50 mM ammonium bicarbonate and 10 mM dithiothreitol followed by reduction at 80°C for 20 min and alkylation with 25 mM iodoacetamide at room temp for 30 min. Proteins were digested with 37.5 μg Worthington modified bovine trypsin for 4 h at 37°C followed by the addition of 1.5% (v/v) trifluoracetic acid and 2% (v/v) acetonitrile and Massprep ADH1 peptide standard (Waters; final concentration 30 fmol ADH1/µl). The SDC precipitate was filtered under vacuum using an ISOLUTE Filtration + filter plate (Biotage) and collected in a Deepwell 96/1,000 µL plate (Eppendorf).

Twelve microliters of peptide digest (∼40 μg) were analyzed per sample in a block-randomized order, interspersed with replicates of a study pool QC (SPQC) sample made from a mixture of all samples. Liquid chromatography used a 1 mm × 150 mm 1.7 µm CSH C18 column (Waters) and a ACQUITY UPLC (Waters) at a flow rate of 100 μL/min and a gradient of 3%–28% acetonitrile over 60 min. The LC was interfaced to a Thermo HF-X MS via a heated electrospray ionization (HESI) source. DIA mass spectrometry analysis used a 120,000 resolution precursor ion (MS1) scan from 375 to 1,500 m/z, an AGC target of 3E6, and maximum injection time (IT) of 20 ms; data were collected in profile mode. MS/MS was performed using 30,000 resolution, an AGC target of 3e6 and maximum IT of 60 ms, and 19 variable DIA windows that spanned 375–1,200 m/z. Data were collected in profile mode.

In addition, ∼800 μg of the SPQC sample was analyzed using high pH-reversed phase (HPRP) fractionation using a 2.1 mm × 5 cm BEH C18 column (Waters) and Agilent 1100 HPLC as previously described (Glisinski et al., 2020) with a mobile phase of 20 mM ammonium formate, pH 10, and a gradient of 3%–35% MeCN over 50 min. Forty-eight equal fractions were collected and concatenated to 12 fractions. After lyophilization, samples were resuspended in 24 μL of 1% (v/v) TFA/2% (v/v) MeCN in H_2_O, and 12 μL was analyzed by microflow-DDA using the same LC method as previously described and a 120,000 resolution precursor (MS1) scan from 375 to 1,500 m/z, an AGC target of 3e6, and maximum injection time (IT) of 45 ms, followed by MS/MS (MS2) of the top 10 most abundant ions at 45,000 resolution with an AGC target of 5e4 ions and 86 milliseconds (ms) IT, 1.2 m/z isolation window, a stepped normalized collision energy (NCE) of 25, 27.5, and 30, a minimum AGC target of 1e4, and 20 s dynamic exclusion.

Data analysis used Spectronaut 13 (Biognosys). A “hybrid” spectral library (Muntel et al., 2019) was generated from DDA and direct-DIA searches which used a UniProt database with *Mus musculus* specificity (downloaded on 04/22/19) appended with sequences for yeast ADH1 and bovine cationic trypsin (17,455 total entries). Default settings were used except for the following: no variable modifications and semitryptic peptide N-termini specificity. DIA files were converted to *.htrms format using a HTRMS converter (Biognosys). Default quantification settings were used except that the workflow used indexed retention time profiling of non-identified precursors; options to carryover peak boundaries and unify peptides were selected. For statistical analysis, single hit proteins were excluded, and quantification was performed at MS2 level using summed precursor areas for the top 20 peptides, Q-value percentile (0.3), and cross-run normalization using q-value complete and median global normalization settings.

### Bioinformatics analysis

#### Data filtering

Twenty-two human keratin proteins were excluded from the heart dataset as they were suspected to be contaminants. In addition, two viral proteins (P31748-Murine leukemia virus and P0C6X9-Murine coronavirus) were removed from the analysis. The list of proteins that were excluded are shown in Supplementary Table S9.

### Statistical analysis of proteomics data

Proteomics datasets underwent sample loading (SL) and trimmed mean of M values (TMM) normalizations procedures (Robinson and Oshlack, 2010) to correct for levels of proteins and compositional bias across samples. In addition, a batch correction was performed for the heart data guided by QC results using the ComBat procedures (Johnson et al., 2007). Following this, differentially expressed proteins (DEPs) were identified for irradiated versus sham irradiated samples in the heart and plasma data using exact test in edgeR (Robinson et al., 2010). DEPs were filtered for significance using a fold-change ≥ |1.2| and a *p*-value of ≤0.05. For the phosphoproteomics data, protocols in PhosR package (Kim et al., 2021; Kim et al., 2021) were used to identify differential phosphosites, kinase-substrate regulatory interactions, and signaling networks.

### Protein orthology mapping, pathway enrichment analysis, and clustering

Mouse proteins were mapped to their corresponding human orthologs using biomaRt (Durinck et al., 2009) and the Mouse Genome Database (Blake et al., 2021). Then, the results of the ortholog mapping were used as an input to the pathway enrichment analysis pipeline. A Gene Set Enrichment Analysis (GSEA) technique (Subramanian et al., 2005) was then used to assess the perturbation of canonical pathways from Biocarta (BIC), KEGG, WikiPathways (WP), Reactome (RECT), and the Pathway Interaction Database (PID). Finally, pathway enrichment maps were utilized to identify interacting pathways (modules) that might have broader impact in the two heart and plasma proteomics data sets. Enrichment map (Merico et al., 2010) in Cytoscape was used for visualizing the network of pathways. For the phosphoproteomics data, phosphosite (or gene) set enrichment analysis was conducted using the Wilcoxon rank sum test as implemented in the PhosR package. Clustering heatmaps were generated using the coolmap function in the Linear Models for Microarray Data (LIMMA) R package (Ritchie et al., 2015).

### Western blot

Mouse heart tissues were mechanically homogenized in Tris Buffered Saline with Tween 20 (TBST) lysis buffer. Protein concentration was determined using BCA assay (Thermo Fisher Scientific #23227, United States) using BioTek Gene 5 software with a 562 nm wavelength. Then, 7.5% precast polyacrylamide gels (Bio-Rad #4568026, United States) were run at 100 V for 75 min and transferred to 0.2 μm Polyvinylidene difluoride (PVDF) membranes by electrophoretic transfer through a tank transfer system at a constant voltage of 30 V for 30 min at 4°C. The membrane was blocked overnight with TBST with 5% non-fat milk (w/v). Primary antibodies KRT10 (Thermo Fisher: MA5-32183, United States), KRT1 (Thermo Fisher: 16848-1-AP, United States) KRT6a (Thermo Fisher: 10590-1-AP, United States), MRPL49 (Thermo Fisher: PA5-112889, United States), fibrinogen gamma (Thermo Fisher: PA5-21968, United States), fibrinogen beta (16747-1-AP; Thermo Fisher, United States), fibrinogen alpha (20645-1-AP; Thermo Fisher, United States), calmodulin 2 (PA5-121166, Thermo Fisher, United States), alpha 2 macroglobulin (MA5-38211, Thermo Fisher, United States), and CAMK2G (CAMKII-301AP, Thermo Fisher, United States) were incubated for 2 h in TBST with 5% non-fat milk (w/v). The membranes were washed prior to incubation with secondary antibody Anti-Rabbit HRP conjugated Ab from (BioRad: #1706515, United States) for 1 hour and subsequently washed with TBST. Membrane was visualized with Clarity™ Western ECL Substrate (Bio-Rad # 1705060, United States) for 5 min, and chemiluminescent images were taken using a BioRad Chemidoc. Densitometric analysis was conducted with Image Lab 6.1 software (Bio-Rad: #12012931, United States).

### Immunofluorescence staining

Fresh frozen heart bases were embedded in Tissue-Tek® O.C.T. Compound and sectioned to 10 µm. The sections were then fixed with 4% paraformaldehyde and permeabilized with 0.1% Tween-20 (Sigma Aldrich, United States) in phosphate buffered saline (PBS). They were then blocked with 5% normal goat serum in 0.1% Tween-20. The primary antibodies used were Fibrinogen beta (16747-1-AP; Thermo Fisher, United States), Fibrinogen alpha (20645-1-AP; Thermo Fisher, United States) C4b-BP (PIPA528017; Thermo Fisher, United States), and C4d (HP8033; Hycult Biotech, United States) each at a final dilution of 1:200. After washing, they were incubated with polyclonal goat anti-rabbit IgG Alexa Fluor 594 conjugated antibody (ab150080; Abcam, United States) at a final dilution of 1:400. ProLong Gold Antifade Mountant with DAPI (Invitrogen, United States) was added onto the section and a coverslip was placed. A Leica 780 Upright Microscope (Wetzlar, Germany) was used to acquire the images of the tissue sections.

### Immunofluorescence staining for neutrophil extracellular traps

Frozen sections of 10 μm, taken from the cardiac apex of mice embedded in OCT, were utilized to investigate the presentation of neutrophil extracellular traps (NETs). To stain the samples, a rabbit anti-elastase antibody (Catalog # PA5-88916, Thermo Fisher, Waltham, MA) diluted 1:100 and a goat anti-myeloperoxidase (MPO) primary antibody (AF 3667, R and D Systems, Minneapolis, MN) diluted to a concentration of 10 μg/mL were employed. Subsequently, the sections were treated with a goat anti-rabbit secondary polyclonal antibody conjugated to Alexa Fluor 594 (Abcam, Cat no. ab150080, Cambridge, United Kingdom) at a dilution of 1:200 and a donkey anti-goat secondary polyclonal antibody conjugated to Alexa Fluor 488 (Catalog # A-11055; Thermo Fisher, Waltham, MA) at a dilution of 1:200. The formation of NETs was examined using a Nikon Ti2 Eclipse inverted confocal microscope.

### Immunohistochemistry for collagen types I and III

Frozen sections of the cardiac apex were treated with anti-COL1A1 antibody (Catalog 72026; CST; United States) at a dilution of 1:100 and with anti-type III collagen antibody (Catalog # PA5-3478716, Thermo Fisher, Waltham, MA) at a dilution of 1:150. This was done after the sections underwent a sequence of processing steps: fixation in 4% paraformaldehyde, peroxidase blocking, antigen retrieval, and protein blocking. A horse polyclonal anti-rabbit secondary antibody from the ImmPress HRP Polymer Detection Kit was subsequently applied. These sections were then developed using a 3,3′-diaminobenzidine solution, followed by counterstaining with hematoxylin. After dehydration, clearing, and mounting, serial sections stained for collagen I or III were captured using a digital camera on a Zeiss Axio Imager Z2 Upright Microscope. The stained regions were analyzed using ImageJ software, with five different viewpoints taken from each section. Results are presented as the percentage of the total area for both collagen types I and III.

## Results

### Cardiac and plasma protein abundance and phosphorylation are modestly diminished at 9 months post GCR_5-ion_ exposure

For the heart proteome, there were 87 significant DEPs (Supplementary Table S1); for the heart phosphoproteome, there were 60 significant differentially phosphorylated peptides (Supplementary Table S3); and for the plasma proteome, there was only one upregulated protein (Supplementary Table S2).

**FIGURE 2:**
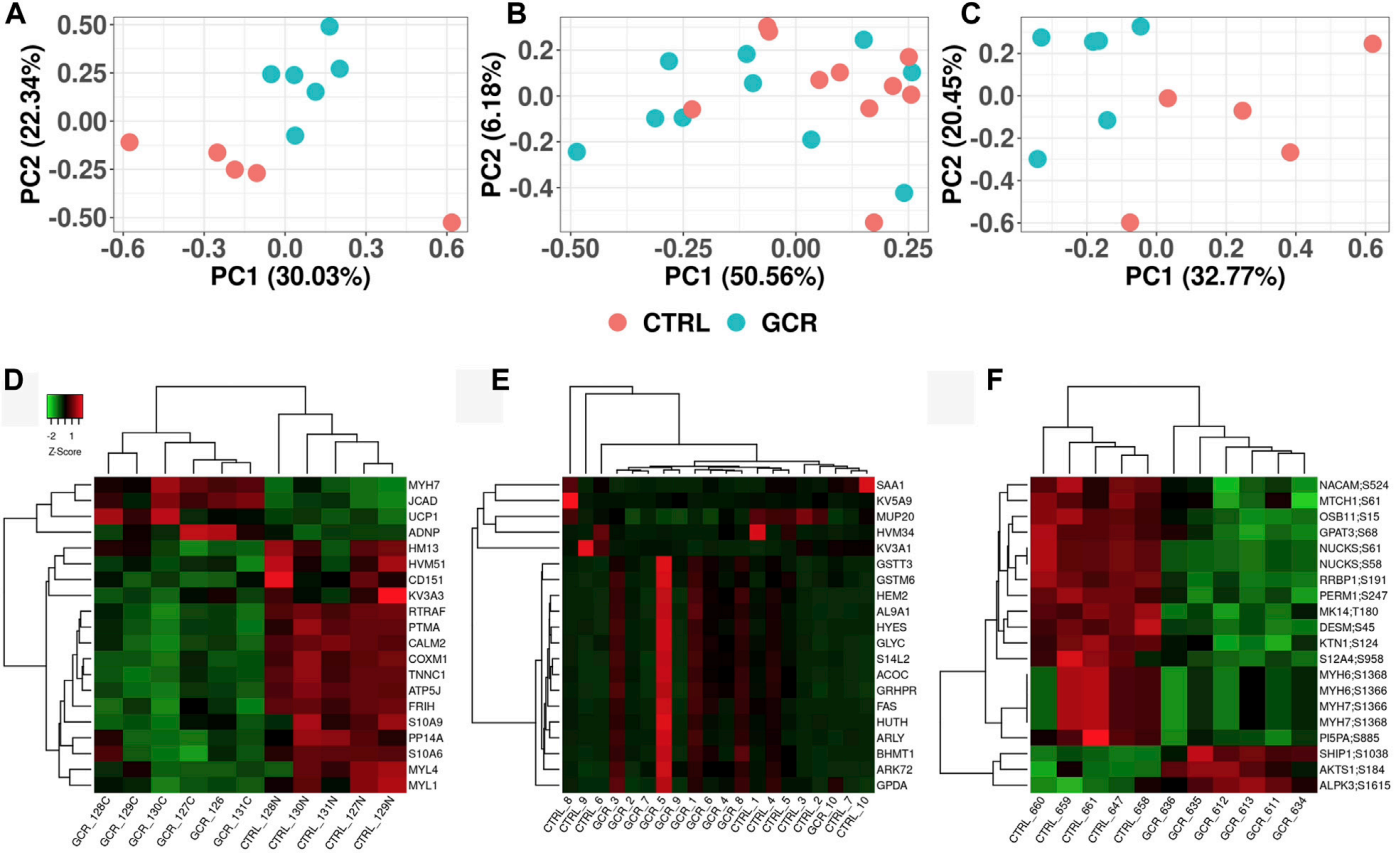
Visualization of proteome and phosphoproteome data sets Principal component analysis (PCA) plots of heart proteome **(A)**; plasma proteome **(B)**; and phosphoproteome **(C)**. Heatmap clustering plot of heart proteome **(D)**; plasma proteome **(E)**; and phosphoproteome **(F)**. Clustering of top 20 proteins in each data set. Proteins are selected based on fold-change difference between GCR_5-ion_ vs. control samples.

Principal components analysis (PCA) was performed in order to observe any high-level differences between sample groups (Figures 2A–C). The heart proteome (Figure 2A) and heart phosphoproteome (Figure 2C) were better separated (sham irradiated versus GCR_5-ion_ irradiated) as judged by the two most prominent components (PC1 versus PC2), whereas the plasma proteome from the sham and GCR_5-ion_ irradiated animals were not as well separated by PC1 and PC2 (Figure 2B).

From the list of significantly changed proteins, we chose the top 20 based on fold change values to create a heatmap as shown in Figures 2D, E (see also Tables 1–3 for more details). The top three DEPs in the cardiac proteome were ATP synthase-coupling factor 6, mitochondrial (ATP5J; AdjPval = 1.53E-28), Troponin C, slow skeletal and cardiac muscles (TNNC1; AdjPval 5.20E-27), and Calmodulin-2 (CALM2; AdjPval = 7.44E-16). The 20 most significant DEPs in the cardiac proteome (Table 1) are involved in the innate and adaptive immune responses, complement activation, calmodulin binding, mitochondrial function, and cardiac contraction. Interestingly, there were two S100 proteins (S100-A6 and S100-A9) in the top cardiac proteome DEPs.

**TABLE 1:** Top 20 proteins: cardiac proteome.

Protein name	Log fold change	*p*-value	Protein description	Function
MYH7	0.7489	7.99E-08	Myosin heavy chain 7	Actin filament binding; calmodulin binding
JCAD	0.4211	1.72E-05	Junctional protein associated with coronary artery disease (JCAD)	Cell adhesin; cell-cell junctions
UCP1	0.6401	2.40E-03	Mitochondrial brown fat uncoupling protein 1	Adaptive thermogenesis; mitochondrial protein
ADNP	1.4620	2.18E-02	Activity-dependent neuroprotector homeobox protein	Activation of protein kinase activity
HM13	−0.5936	4.96E-02	Minor histocompatibility antigen H13	Membrane protein proteolysis involved in retrograde protein transport
HVM51	−0.3970	1.11E-02	Ig heavy chain V region AC38 205.12	Complement activation, innate immune response
CD151	−0.5773	8.84E-03	CD151 antigen (GP27)	Cell migration; positive regulation of cell migration; T cell proliferation
KV3A3	−1.0194	3.52E-02	Ig kappa chain V-III region MOPC 70	Adaptive immune response; immune response
RTRAF	−0.4375	1.39E-14	RNA transcription, translation, and transport factor protein	Negative regulation of protein kinase activity
PTMA	−0.5383	3.36E-12	Prothymosin alpha	Histone exchange; negative regulation of apoptosis
CALM2	−0.4734	7.44E-16	Calmodulin-2	Activation of adenylate cyclase activity
COXM1	−0.4160	2.56E-04	COX assembly mitochondrial protein	Mitochondrion
TNNC1	−0.8406	5.20E-27	Troponin C, slow skeletal and cardiac muscles	Cardiac muscle contraction
ATP5J	−0.6049	1.53E-28	ATP synthase-coupling factor 6, mitochondrial	ATP metabolic process
FRIH	−0.4268	7.79E-11	Ferritin heavy chain	Immune response; intracellular sequestering of iron ion
S10A9	−0.6228	1.13E-03	Protein S100-A9	Actin cytoskeleton reorganization
PP14A	−0.4267	1.10E-03	Protein phosphatase 1 regulatory subunit 14A	Regulation of phosphorylation
S10A6	−0.4436	2.24E-03	Protein S100-A6	Calcium-dependent protein binding
MYL4	−0.4648	8.34E-03	Myosin light chain 4	Cardiac muscle contraction
MYL1	−0.4847	9.36E-05	Myosin light chain 1/3	Cardiac muscle contraction

For the plasma samples, there were three perturbed proteins of note; major urinary protein 20 (MUP20; AdjPval = 2.80E-03), Serum amyloid A-1 protein (SAA1; AdjPval = 9.25E-02), and Glutathione S-transferase theta-3 (GSTT3; AdjPval = 9.25E-02). The top 20 proteins in the plasma proteome are involved in complement activation, innate and adaptive immune responses, biosynthetic and metabolomic processes, and acute phase responses (Table 2).

**TABLE 2:** Top 20 plasma proteome proteins.

Protein name	Log fold change	*p*-value	Protein description	Function
ACOC1	−1.3977	3.27E-02	Aconitase	Cellular iron homeostasis
ARK72	−1.5265	1.82E-02	Aflatoxin B1 aldehyde reductase member 2	Doxorubicin metabolic processes
GLYC	−1.6638	3.79E-02	Serine hydroxymethyltransferase	Serine and glycine biosynthetic processes
MUP20	1.1216	5.92E-06	Major urinary protein 20	Olfactory learning
SAA1	1.5990	6.61E-04	Serum amyloid A-1 protein	Acute-phase response; cholesterol metabolic process
HVM34	2.5801	2.27E-03	Ig heavy chain V region AMPC1	Complement activation; innate immune response
KV5A9	1.5679	3.18E-03	Ig kappa chain V-V region L7 (Fragment)	Adaptive immune response
KV3A1	1.3757	5.73E-03	Ig kappa chain V-III region PC 2880/PC 1,229	Adaptive immune response
HYES	−1.6366	6.40E-03	Bifunctional epoxide hydrolase 2	Cholesterol homeostasis; production involved in inflammatory response
ARLY	−1.6800	6.06E-03	Argininosuccinate lyase (ASAL)	Arginine biosynthetic process via ornithine
AL9A1	−1.7514	6.96E-03	4-trimethylaminobutyraldehyde dehydrogenase	Carnitine biosynthetic process
FAS	−1.4609	7.53E-03	Fatty acid synthase	Acetyl-CoA metabolic process
BHMT1	−1.5636	6.06E-03	Betaine-homocysteine S-methyltransferase 1	Amino-acid betaine catabolic process
GPDA	−1.3218	3.06E-2	glycerol-3-phosphate dehydrogenase	Carbohydrate metabolism; gluconeogenesis
GSTT3	−1.4132	4.09E-04	Glutathione S-transferase theta-3	Glutathione metabolic process
GSTM6	−2.1219	1.51E-02	Glutathione S-transferase Mu 6	Glutathione metabolic process
GRHPR	−1.3113	2.02E-02	Glyoxylate reductase	Dicarboxylic acid metabolic process
HEM2	−1.5299	1.58E-02	Delta-aminolevulinic acid dehydratase	Cellular response to ions (lead, aluminum, cadmium)
HUTH	−1.8821	9.97E-03	Histidine ammonia-lyase (Histidase)	Histidine catabolic process
S14L2	−1.3317	1.33E-02	SEC14-like protein 2	Acute phase response

Proteins of note in the phosphoprotein data set are: MYH7 (myosin 7); RRBP1 (ribosomal-binding protein 1); SHIP1 (phosphatidylinositol 3, 4, 5-trisphosphate 5-phosphatase 1); PI5PA (phosphatidylinositol 4,5 -bisphosphate 5-phosphotase A); NUCKS (nuclear ubiquitous casein and cyclin-dependent kinase substrate 1); LAPR1 (La-related protein 1); DESM (desmin); and AKTS1 (AKT1 substrate 1). The top 20 proteins in the cardiac phosphoproteome are involved in cardiac function, enzymatic activity, muscle contraction, regulation of transcription and translations, DNA repair, and regulation of neutrophil migration (Table 3).

**TABLE 3:** Top 20 phosphopeptides.

Protein name	Log fold change	Adjusted *p*-value	Phosphopeptide site; sequence	Protein function
ALPK3; Alpha kinase 3	0.5840	2.15E-02	S1615; GSKSPSAGR	Hypertrophic cardiomyopathy; protein serine kinase
DESM; desmin	−0.4916	1.94E-02	S45; AGFGTKGSSSSMTSR	Inter-connects the Z disks of sarcomeres
GPAT3; Glycerol-3-phosphate acyltransferase 3	−0.4454	4.51E-02	S68; NSASVGIIQR	Enzymatic activity
KTN1; Kinectin	−0.4739	4.03E-02	S124; QKPSLEEQVIK	ER protein; involved in kinesin-driven motility
MTCH1; Mitochondrial carrier homolog 1	−0.5770	4.51E-02	S61; HPRPAAQPSAR	Mediates insertion of transmembrane proteins into the mitochondrial outer membrane
JPH2; Junctophilin-2	−0.4402	1.20E-02	T621; QATLEPKPIVPK	Provides structural bridge between plasma membrane and SR; required for excitation-contraction coupling in cardiomyocytes
MYH6; Myosin 6	−0.5386	3.44E-02	S1368; VLSKANSEVAQWR	Muscle contraction
MYH7; Myosin 7	−0.5386	3.44E-02	S1366; VLSKANSEVAQWR	Muscle contraction
LARP1; La-related protein 1	0.4357	1.29E-02	S743: SLPTTVPESPNYR	Regulates translation of specific mRNA downstream of mTORC1 complex
NACAM; Nascent polypeptide-associated complex subunit alpha, muscle-specific form	−0.4512	8.19E-03	S524; ESPSSQSASSLEVLSEDTVTK	Cardiac and muscle specific transcription factor
NUCKS; Nuclear ubiquitous casein and cyclin-dependent kinase substrate 1	−0.5010	7.713E-03	S58; NSQEDSEDSEEKDVK	DNA repair by promoting homologous recombination
NUCKS; Nuclear ubiquitous casein and cyclin-dependent kinase substrate 1	−0.5010	7.713E-03	S61; NSQEDSEDSEEKDVK	DNA repair by promoting homologous recombination
OSB11; Oxysterol-binding protein-related protein 11	−0.4590	6.85E-03	S15; VSESEGKLEGLATAVTPNK	Regulates FABP4 levels in differentiating adipocytes
PERM1; PGC-1 and ERR-induced regulator in muscle protein 1	−0.5430	1.09E-02	S247; EESGLDLSTPILITEQDQIRK	Regulates expression of selective PPARGC1 A/B and ESRRA/B/G target genes in glucose and lipid metabolism, energy transfer, and contractile function
PI5PA; Phosphatidylinositol 4,5 -bisphosphate 5-phopshatase A	−0.4498	4.51E-02	S885; SRSPGLAR	Modulates function of inositol and phosphatidylinositol polyphosphate-binding proteins that are present in membrane ruffles
RRBP1; Ribosome-binding protein 1	−0.5006	2.74E-03	S191; ASSPATSSQGK	Mediates interaction between the ribosome and the ER membrane
S12A4; Solute carrier family 12 member 4	−0.4770	1.34E-02	S958; DRHSALR	Mediates potassium-chloride cotransport
AKTS1; AKT1 substrate 1	0.7830	3.44E-02	S184; SLPVSVPVWAFK	Subunit of mTORC1
SHIP1; Phosphatidylinositol 3, 4, 5-trisphosphate 5-phosphatase 1	0.4719	8.18E-03	S1038; KEQESPK	Key regulator of neutrophil migration
MK14; Mitogen-activated protein 14	−0.6623	8.11E-03	T180; HTDDEMTGYVATR	Serine/threonine protein kinase

**FIGURE 3:**
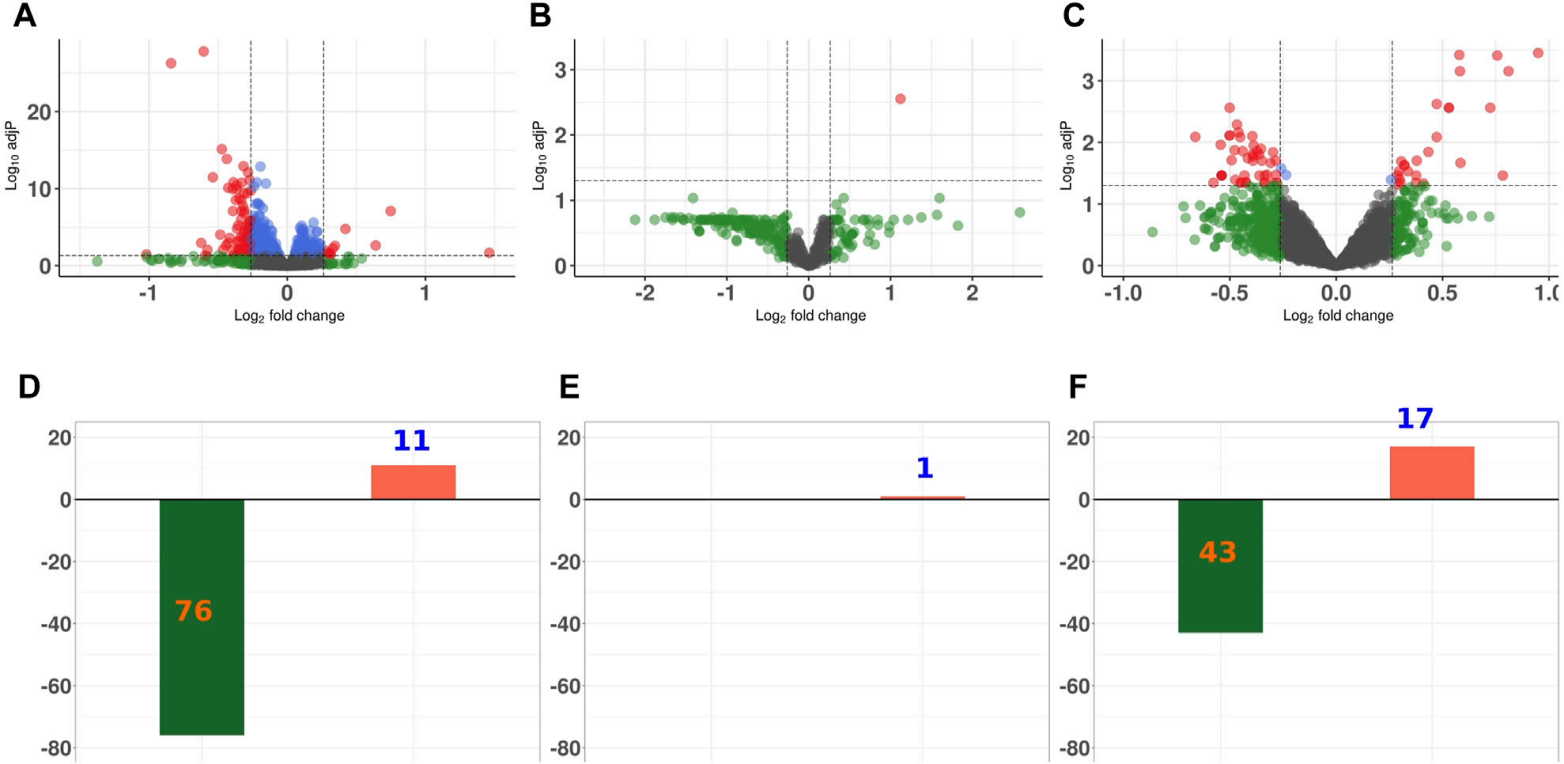
Differential expression analysis of proteins in proteome and phosphoproteome data sets Volcano plot representing the overall perturbation significance of proteins based on *p*-values and fold-change criteria for heart proteome **(A)**; plasma proteomc **(B)**; and phosphoproteome **(C)**. Each dot represents one protein. Log2FC indicates the mean expression level for each protein. Black dots represent non-significant proteins; green dots represent proteins that have a FC ≥ 1.2; blue dots represent proteins that have a *p*-value ≤0.05; and orange dots represent those proteins with a *p*-value of ≤0.05 and FC ≥ 1.2. Bar graph showing the number of significantly up- and downregulated proteins for heart proteome **(D)**; plasma proteome **(E)**; and phosphoproteomc **(F)**.

For all three proteomics data sets, the majority of the DEPS were diminished in abundance in GCR_5-ion_ irradiated versus sham irradiated control animals (Figure 3). In the proteome of the heart, there were 76 proteins that were significantly diminished in abundance and 11 proteins that were significantly increased in abundance. In the plasma proteome, there was only one protein with increased abundance after radiation. In the phosphopeptide data set, there were 43 phosphopeptides that were decreased in abundance after radiation and 17 phosphopeptides that increased post radiation.

### A single dose of GCR_5-ion_ permanently changed numerous biological pathways in the heart and plasma

**FIGURE 4:**
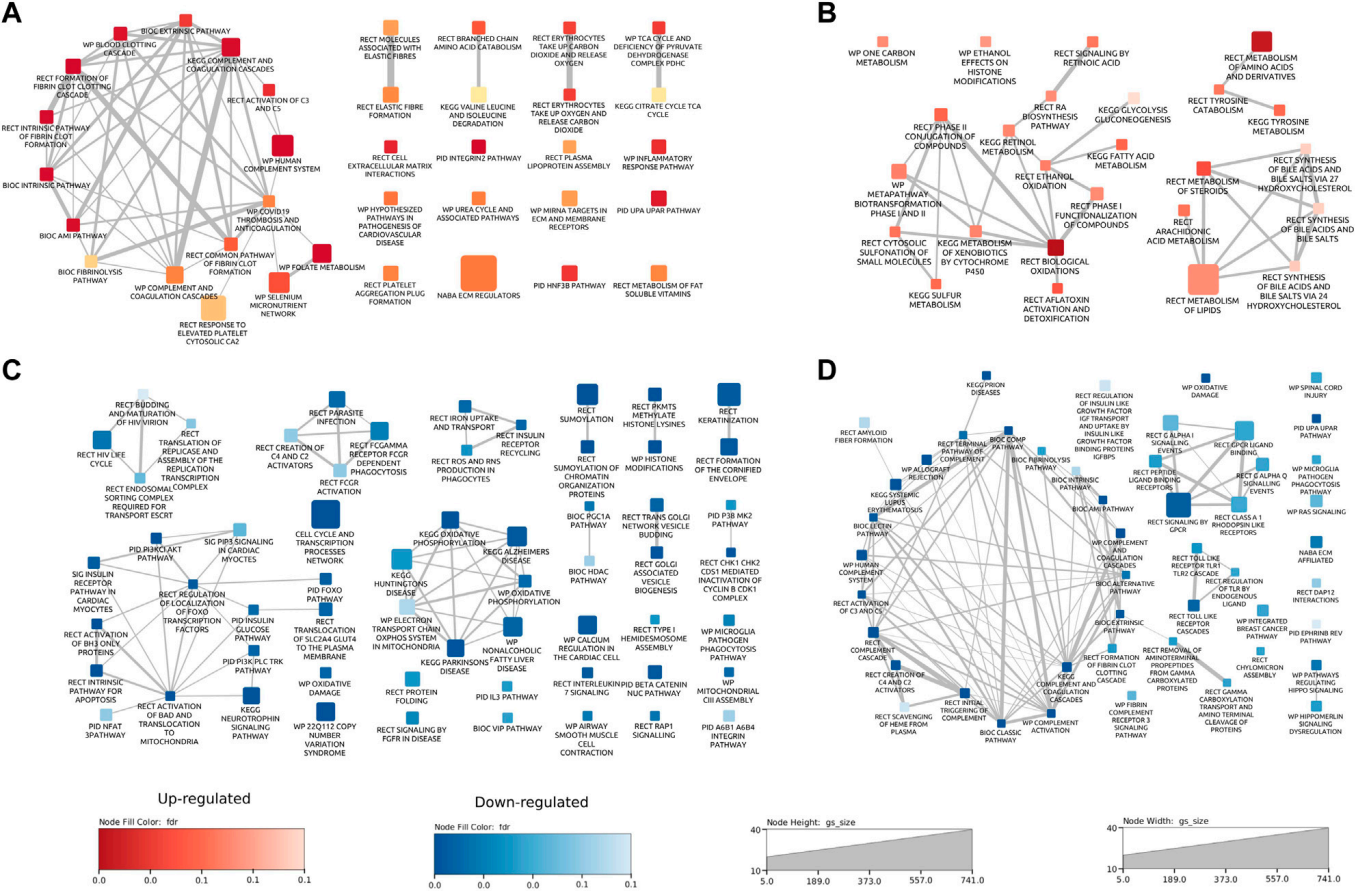
Pathway enrichment maps for proteome data sets. Pathway interaction plots for upregulated [**(A)**; red] and downregulated [**(C)**; blue] pathways in heart proteome data. Pathway interaction plots for upregulated [**(B)**; red] and downregulated [**(D)**; blue] pathways in plasma proteome data. Each rectangle represents a pathway. The size of the rectangle is proportional to the number of proteins annotated with the pathway. Two interacting pathways are connected by a line. The thickness of the line is proportional to the number of proteins shared between interacting pathways. Pathways are annotated with the source database as Biocarta (BIC), Kyoto Encyclopedia of Genes and Genomes (KEGG), WikiPathways (WP), Reactome (RECT), and the Pathway Interaction Database (PID).

The results from both the abundance and phosphoproteomics data sets indicated that there were a number of proteins significantly changed in abundance in response to GCR_5-ion_. Each data set was analyzed using a bioinformatics/gene functional-enrichment approach, as outlined in the Materials and Methods, to map these changes to cellular location, protein function, and biological pathways. For the heart proteome, 166 pathways were altered (36 upregulated and 130 downregulated); for the plasma proteome, there were 73 pathways altered (25 upregulated and 48 downregulated); and for the phosphoproteome, there were 223 pathways affected at 0.1 FDR cutoff. See Supplementary Table S4–S8 for details on all pathways emerging from the bioinformatics/gene functional-enrichment approach. For the heart proteome, the major upregulated enriched pathways were: inflammatory processes (including coagulation cascade, complement cascade), elastic fibers, amino acid metabolism, citric acid cycle, extracellular matrix interactions, integrins, cardiovascular disease pathogenesis, urea cycle, HNF-3B/FOXA2 pathway, and metabolism of fat-soluble vitamins (Figure 4A). Major enriched pathways that were downregulated in the heart proteome were: cell cycle/transcription (CCTR), HIV life cycle, iron uptake and transport, reactive oxygen species, apoptosis, insulin glucose pathways, mitochondrial functions (including oxidative phosphorylation and electron transport system), and keratinization/cornification (Figure 4C). The CCTR pathway contained the most proteins and Supplementary Figure S1 shows the complete pathway interaction maps for CCTR. Each of the 72 circles represents a pathway in the interaction map with the size of the circle proportional to the number of proteins annotated with each pathway. Two interacting pathways are connected by a line with the thickness of the line proportional to the number of proteins shared between interacting pathways.

Major enriched pathways that were upregulated in the plasma samples from mice exposed to GCR_5-ion_ included oxidative damage, and metabolism of amino acids, fatty acids, lipids, toxins, and steroids, toxins (Figure 4B). Major enriched pathways that were downregulated in the plasma include inflammatory processes (complement cascade, coagulation), oxidative damage, and cardiac signaling (GPCR and Hippo) (Figure 4D).

**FIGURE 5:**
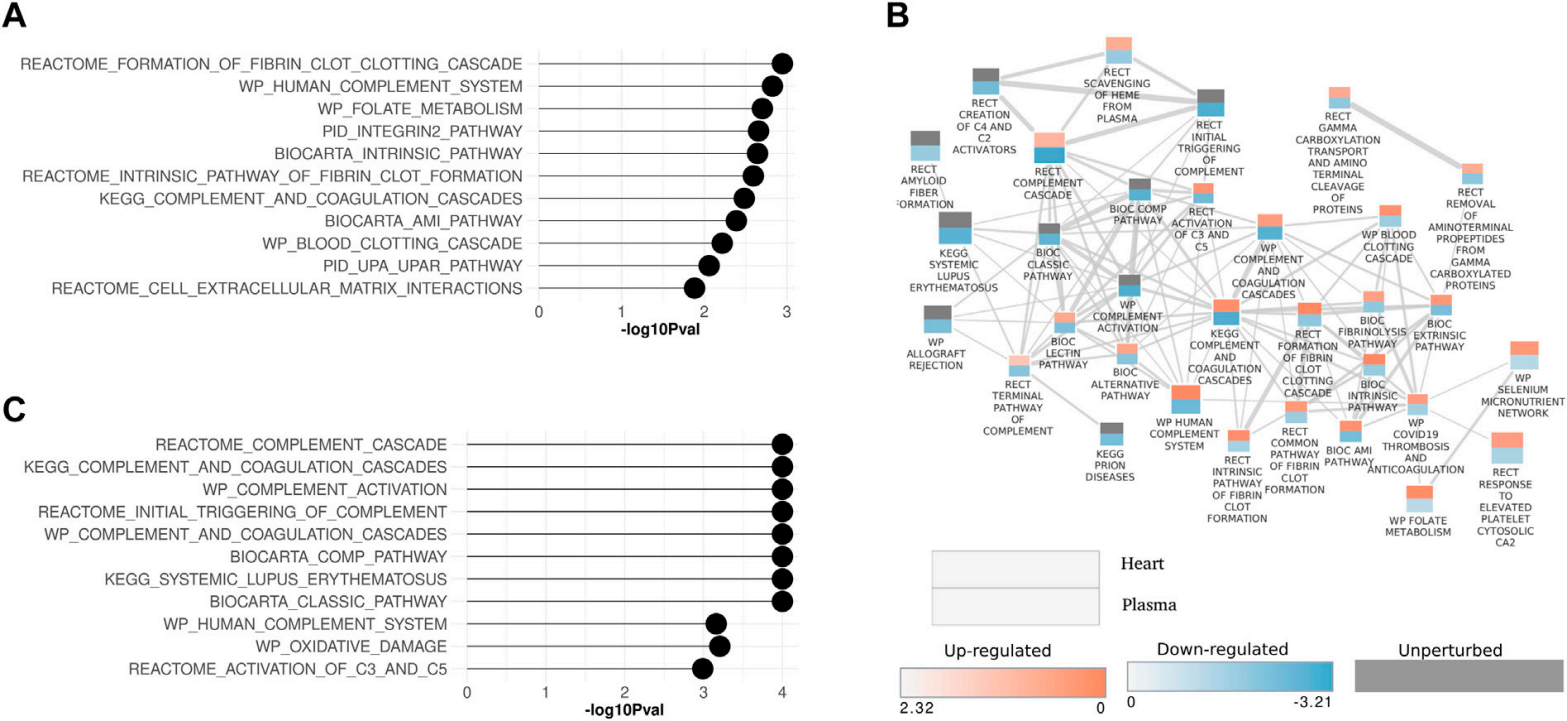
Enrichment maps for inflammation-related pathways in heart and plasma proteome data sets **(A)** Lollipop plot showing top ten significantly modulated inflammation pathways in the heart and **(C)** plasma proteome. **(B)** Pathway enrichment map showing inflammation pathways that are perturbed in common in the heart and plasma proteome data sets. Each rectangle represents a pathway. The rectangle is split in two. The upper portion of the rectangle represents the heart data and lower half represents the plasma data. The size of the rectangle is proportional to the number of proteins annotated with the pathway. Two interacting pathways are connected by a line. The thickness of the line is proportional to the number of proteins shared between interacting pathways. Pathways are annotated with the source database as Biocarta (BIC), Kyoto Encyclopedia of Genes and Genomes (KEGG), WikiPathways (WP), Reactome (RECT), and the Pathway Interaction Database (PID).

Inflammatory pathways and processes that were perturbed in both the heart and plasma proteome are examined in more detail in Figure 5. In both the heart and plasma proteome data set, pathways related to inflammatory processes were in the top ten significantly modulated pathways of each (Figures 5A, C). A pathway enrichment map combining both data sets with a focus on the inflammation pathways was generated (Figure 5B). Interestingly, two patterns emerged from this analysis. First, for pathways that were commonly perturbed in the plasma and heart, the pathways were upregulated in the heart and downregulated in the plasma, as shown by the red top half representing the heart and the bottom blue half that representing the plasma. Second, inflammatory pathways that were only perturbed in the plasma were downregulated.

**FIGURE 6:**
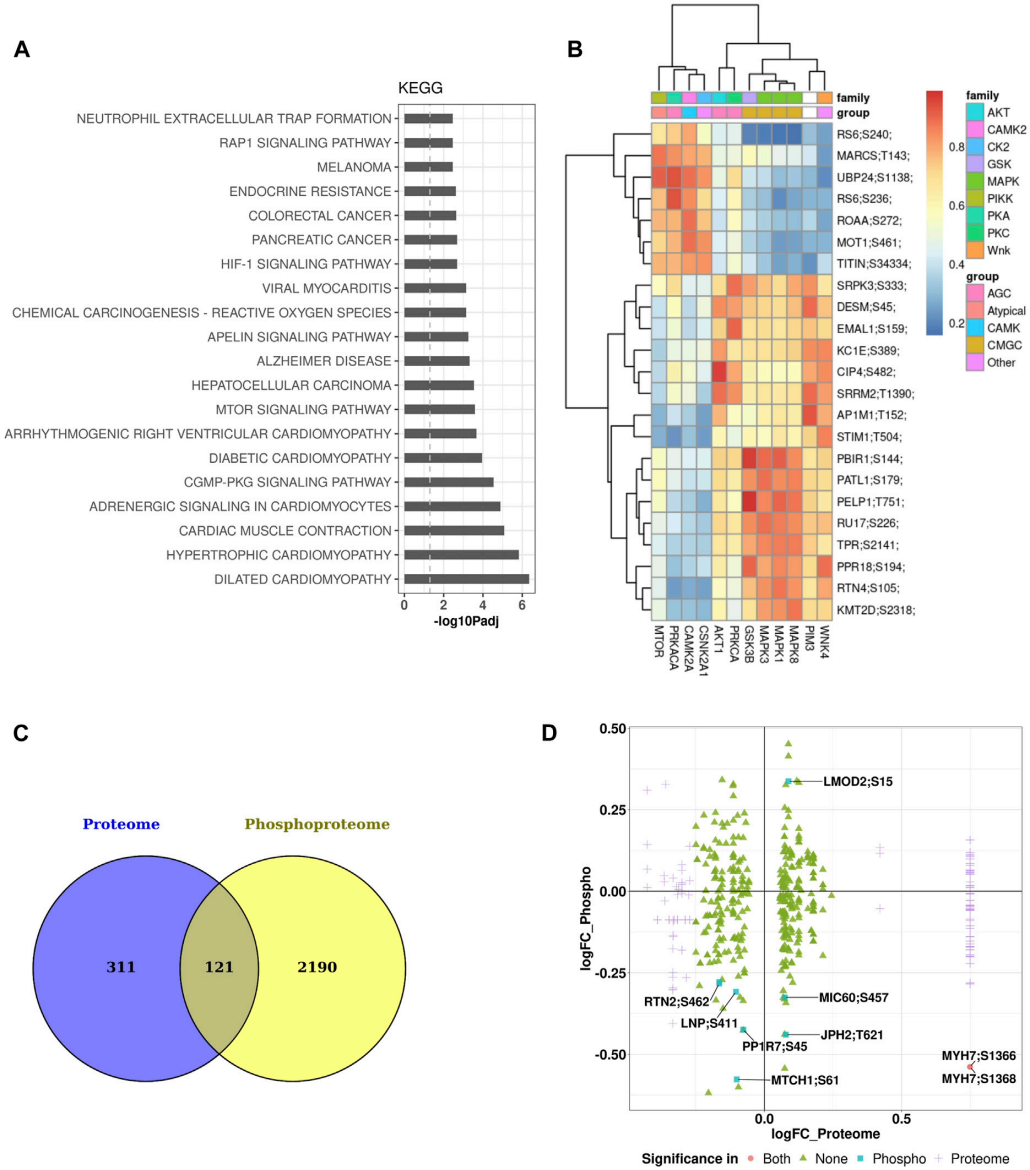
Pathway enrichment and kinome maps for phosphoproteome data **(A)** Significantly perturbed pathways in GCR irradiated samples compared to control. Top 20 over-represented pathways from the Kyoto Encyclopedia of Genes and Genomes (KEGG). **(B)** Heatmap of kinase-substrate interaction (kinome map). **(C)** Venn diagram showing overlap of differentially expressed proteins among heart proteome and phosphoproteome. **(D)** Plot of log fold change (FC) of proteome versus log FC of phosphoproteome for 121 overlapping proteins in the two data sets.

Major enriched pathways that were significantly modulated in the phosphoproteomics data set of the heart included many pathways and processes involved in cardiac structure and function such as sarcomere, contractile fiber, I band, muscle cell development, contraction, actin binding, cardiac differentiation, and transmembrane transport. Other pathways significantly perturbed involved inflammatory processes such as neutrophil extracellular trap formation and reactive oxygen species (Figure 6A). Kinases responsible for phosphorylation of major substrates are shown in the heatmap of kinase-substrate interaction (Figure 6B). Notably, MTOR, CAMKG2, and PIM3 emerged as the three kinases responsible for phosphorylating most of the major substrates examined in the analysis. There were 121 proteins that are common between the heart proteome and phosphoproteome (Figure 6C). Of these proteins, there were seven phosphopeptides that were significantly affected in only the phosphoprotome data set (Figure 6D), indicating that the differences in phosphopetpide abundance of these seven phosphopepitides were due to differential phosphorylation at these particular sites rather than differences in protein abundance. These proteins are: RTN2 (reticulon 2), LMOD2 (leiomodin 2), PP1R7 (protein phosphatase 1 regulatory subunit 7), MTCH1 (mitochondrial carrier 1), LNP (lunapark), MIC60 (MICOS complex subunit Mic60), and JPH2 (Junctophilin-2).

### Neutrophil extracellular traps were elevated in myocardium of GCR_5-ion_ irradiated mice 12 months post irradiation

Western blot analysis was performed on mouse myocardium of 150 cGy GCR_5-ion_ irradiated mice at 12 months and age-matched sham irradiation control (Supplementary Figure S2) to evaluate the expression patterns of selected proteins where antibodies were commercially available. Of the 13 protein targets examined by this methodology, only cytokeratin1 and fibrinogen gamma were statistically significantly different between hearts from irradiated and sham irradiated mice (Supplementary Figure S2D).

Immunofluorescent staining of mouse myocardium for markers of inflammation was also performed. Shown in Supplementary Figure S3 are representative myocardial sections from mice irradiated with 150 cGy GCR_5-ion_ stained for fibrinogen α, fibrinogen β, C4d, and C4b-BP. There was no observed difference in the staining patterns of these protein markers between the GCR_5-ion_ irradiated and sham irradiated mice and there was no significant staining observed in any of the samples.

**FIGURE 7:**
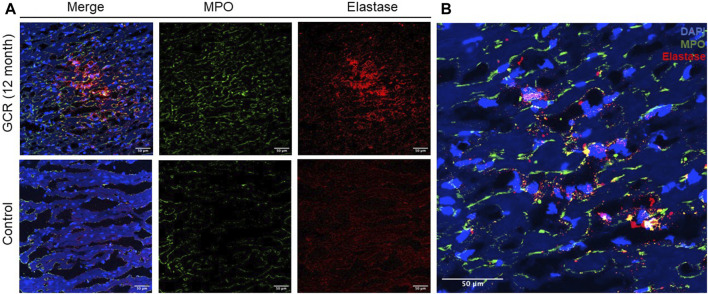
The presence of neutrophil extracellular traps (NETs) within the myocardial tissue was observed in a mouse model exposed to GCR_5-ion_. Immunofluorescence staining was performed to assess the presence of NETs in the hearts of mice exposed to 150 mGy of GCR_5-ion_ at 12 months post radiation, as compared to sham irradiated control mice hearts. **(A)** A robust and extensive distribution of NETs was observed in the myocardium of mice exposed to GCR_5-ion_ (upper panel), while no NETs were detected in the control mice hearts (lower panel). **(B)** The representative images of NETs structure in the hearts of mice exposed to GCR were obtained. The colocalization of extracellular DNA structure (DAPI), MPO, and elastase further confirmed the presence of NET structures **(B)**. The scale bar represents 50 μm.

To investigate the histologic presentation of neutrophil extracellular traps (NETs) in the hearts of mice exposed to GCR_5-ion_, immunofluorescent staining was performed using anti-neutrophil elastase and anti-MPO antibodies. The staining patterns were compared between the GCR_5-ion_ -exposed mice hearts and the hearts of control mice (Figure 7A). The colocalization of DAPI, MPO, and neutrophil elastase revealed positive staining for NETs in the myocardial tissue of GCR_5-ion_ -treated mice (Figure 7B). The NETs were found to be diffusely distributed in these heart sections, whereas no NETs were observed in any of the control mice hearts.

## Discussion

Based on data from epidemiological studies, it takes a significant amount of time for cardiovascular disease to develop (up to decades). There is relatively little knowledge regarding the time frame to develop cardiovascular disease from exposure to the space radiation environment. Here, we undertook a multi-proteomics approach to characterize protein and pathway perturbations that occurred in hearts and plasma from mice exposed to a single dose of GCR_5-ion_ 8 months prior.

One of the most interesting observations was that across the three data sets, the abundance of the majority of the DEPs diminished in the heart and plasma at 8 months post radiation. While one acute effect of radiation is to cause fragmentation and aggregation of protein molecules (Kumta and Tappel, 1961), it is possible that the effects observed at 8 months post radiation may be due to the inhibition of protein synthesis. We previously reported that microgravity, another stressor of space travel, reduced protein turnover in cardiomyocytes (Feger et al., 2016) and speculated that this diminishment in protein synthesis could lead to overall reduction in cardiomyocyte protein content.

Calmodulin 2 (CALM2), a signaling protein involved in the calcium-regulated contraction of cardiac muscle (Beghi et al., 2022), was one of the most highly perturbed proteins in the heart proteome in this study. We examined the expression levels of CALM2 as well as an associated kinase (CAMKG2) via Western blots but did not see a statistical difference in abundance of either protein in the hearts of GCR_5-ion_ irradiated mice compared to unirradiated controls (Supplementary Figures S2A, S3C). Western blotting and immunohistochemistry were also performed on hearts from irradiated and sham irradiated mice to validate markers of complement activation (C4d and C4b-BP; Supplementary Figure S3) and coagulation/clotting/fibrin formation (fibrinogen alpha, fibrinogen beta, fibrinogen gamma; Supplementary Figures S2C, S3). From this analysis, only fibrinogen gamma was statistically significantly different between hearts from irradiated and sham irradiated mice as observed via Western blot analysis (Supplementary Figure S2H).

In the plasma proteome, the most significantly regulated protein was major urinary protein 20 (MUP20) which was upregulated in irradiated animals. MUP20 is a male pheromone which promotes aggressive male behavior, stimulates female sexual attraction to male urinary scent, promotes a strong learned attraction to the airborne urinary odor of an individual male, and promotes spatial learning by rapidly conditioning preference for its remembered location among females and competitor males so that animals prefer to spend time in the site even when scent is absent (Roberts et al., 2010; Roberts et al., 2010; Demir et al., 2020). While this finding may not be translatable since humans are the only placental mammals found not to have any active MUP genes (Logan et al., 2008), the finding of MUP20 dysregulation in male mice exposed to GCR_5-ion_ may illuminate behavioral and learning changes following exposures of rodents to various GCR components.

Other proteins dysregulated in the plasma following GCR irradiation are serum amyloid A1 (SAA1) (upregulated) and glutathione S-transferase theta-3 (GSTT3) (downregulated). SAA1 is a major acute-phase protein mainly produced by hepatocytes in response to infection, tissue injury, and malignancy. SAA has anti-inflammatory properties by inhibiting platelet aggregation and by reducing the oxidative burst in Polymorphonuclear neutrophils(Zimlichman et al., 1990; Gatt et al., 1998). SAA also has proinflammatory effects, such as induction of extracellular matrix degrading enzymes, allowing the repair of tissue damage and inflammatory cytokines such as IL-1B, IL-6, and TNFa (Uhlar and Whitehead, 1999). GSTT3 is expressed strongly in the liver (and to a lesser extent in kidney and testis). Glutathione transferases play a significant role in the metabolism/detoxification process of a wide range of electrophilic compounds that include mutagens, carcinogens, reactive oxygen species, and some therapeutic agents.

The most highly impacted pathways in the heart tissue were inflammation/complement/coagulation, mitochondria, and cell cycle/transcription (CCTR). The downregulation of the mitochondrial pathways is in line with the comprehensive Azimzadeh et al. review of omics data from radiation induced-cardiovascular disease (Azimzadeh et al., 2022). However, in our study, the CCTR cluster was the most highly affected, as detailed in Supplementary Figure S1, which shows the 72 CCTR processes that are downregulated in the heart 8 months post GCR irradiation. This global downregulation in cell cycle and transcription processes may help explain the overall diminishment in protein abundance noted earlier in the discussion.

Perhaps the most interesting finding from this study involves neutrophil extracellular traps (NETs). NETs have come to the forefront of immunology for their fundamental role in combating bacterial pathogens. Beyond this defensive capacity, intriguingly, NETs have shown the ability to modulate inflammatory responses, even within sterile environments (Papayannopoulos, 2018).

One remarkable illustration of this involves the role of cholesterol crystals in prompting the formation of NETs, which subsequently stimulate macrophages to unleash IL-1-beta, a process known to expedite the development of atherosclerosis (Warnatsch et al., 2015). This process becomes particularly concerning when a plaque rupture transpires. The resulting exposure of endovascular NETs can escalate vascular thrombosis, a factor intimately tied to severe cardiovascular events like acute myocardial infarction and stroke (Klopf et al., 2021). Our study has unveiled some illuminating findings in this area. Employing proteomic analysis, we discovered that the NETs formation pathway was activated in mice exposed to Galactic Cosmic Radiation (GCR), and myocardium sections exhibited a significant accumulation of NETs in these mice after long-term high-energy GCR exposure.

Our findings align with previous research wherein, in a mouse bladder cancer model receiving radiation therapy, NETs were found to promote their formation through the protein HMGB1 via a TLR4-dependent pathway both *in vitro* and *in vivo* (Shinde-Jadhav et al., 2021). Notably, the accumulation of NETs and its components in local areas can inflict damage on healthy tissues. Prior research has linked the increased expression of NETs in the inflamed myocardium to gene expression of collagen type I (Col1a1) and increased the heart stiffness in mouse models (Bai et al., 2019). This correlation may explain our previous findings, which demonstrated decreased systolic heart function and increased arterial elastance 1 year after GCR exposure, as revealed through cardiac-pressure-volume loop analysis (Bishawi et al., 2022). From our limited tissue samples, we observed an increased presence of type I collagen in the GCR-exposed mice, but no significant difference in type III collagen between the two groups (see Supplementary Figure S4). Evidence for persistent activity, as indicated by markers such as MPO and elastase, was noted 8 months post-exposure. However, this study did not include additional markers such as plasma enrichment, quantification of NETotic neutrophils, cleaved caspases, and condensed nuclei, which could have provided further insights into the sustained nature of NETs activity. The findings underscore the need for a more comprehensive investigation with an expanded sample size, controlled left ventricular mass data, and methodical sampling at regular intervals throughout the study to evaluate this phenomenon more accurately. NETs have also been shown to promote the development of deep vein thrombosis (DVT) in mouse models (Brill et al., 2012). A complication associated with cardiovascular degeneration during space flight that was recently brought to light is the occurrence of venous thromboembolisms (VTEs). Approximately 2 months into a mission aboard the International Space Station in 2019, a female astronaut noticed an abnormality during an ultrasound examination (Auñón-Chancellor et al., 2020). Upon further examination and consultation with physicians on Earth, the astronaut was diagnosed with a left internal jugular venous thrombosis. The astronaut presented with no signs or symptoms, and she had no family or personal history of venous thromboembolisms. A therapeutic regimen was quickly established for the astronaut and she was luckily treated in a timely manner. This incidence exemplifies the unknown health consequences associated with unique stressors of the space environment. Although there are other confounding factors that may have contributed to the astronaut’s hypercoagulable state, there is evidence that her exposure to space radiation was a primary constituent.

We found NETs presentation in the myocardium, but no significant enrichment of inflammatory cytokines was identified in the plasma. Neutrophils serve as the first-line of innate immunity. The cytokines that trigger NETs formation might be detectable during the acute injury stage. Thus, there is a pressing need for another study that collects samples shortly after GCR exposure to accurately determine what triggers NETs.

While the exact mechanism behind NET formation following GCR exposure remains elusive in our study and whether NET formation may have played a role in the astronaut’s development of VTE is unknown, we propose a hypothesis involving chronic tissue damage and reactive oxygen species (ROS) activation post-radiation exposure as potential mechanisms, warranting further investigation.

## Data Availability

The original contributions presented in the study are publicly available. This data can be found here: massive.ucsd.edu, MSV000092395.

## Supplementary material

SUPPLEMENTARY FIGURE S1 Pathway interaction maps for cell cycle and transcription module Interaction of pathways related to cell cycle and transcription (CCTR) processes that are downregulated in the heart proteome data. Each circle represents a CCTR pathway. The size of the circle is proportional to the number of proteins annotated with the pathway. Two interacting pathways are connected by a line. The thickness of the line is proportional to the number of proteins shared between interacting pathways. Pathways are annotated with the source database as Biocarta (BIC), Kyoto Encyclopedia of Genes and Genomes (KEGG), WikiPathways (WP), Reactome (RECT), and the Pathway Interaction Database (PID).

SUPPLEMENTARY FIGURE S2 Western blot and densitometric analysis of 150 cGy GCR_5-ion_ irradiated mice at 12 months and age-matched sham irradiation control. Calmodulin-2 **(A)**, Alpha 2-Macroglobulin **(B)**, Calcium/calmodulin-dependent Protein Kinase II gamma **(C)**, Cytokeratin 1 **(D)**, Cytokeratin 10 **(E)**, Cytokeratin 6a **(F)**, MRPL 49 **(G)**, Fibrinogen Gamma **(H)**, Fibrinogen Beta **(I)**, Fibrinogen Alpha **(J)**, 14–3-3 Gamma **(K)**, 14-3–3 Epsilon **(L)**, and 14–3-3 Beta **(M)**. A-C: n = 6 each for GCR_5-ion_ and controls; **(D-F,H-M)** n = 4 for GCR_5-ion_ and n = 3 for controls; **(G)** n = 4 each for GCR_5-ion_ and controls. Non-parametric Mann-Whitney test, 2 sided. **p* ≤ 0.05, ***p* ≤ 0.01.

SUPPLEMENTARY FIGURE S3 Immunofluorescent staining of mouse myocardium for markers of inflammation. Representative myocardial sections from mice irradiated with 150 cGy GCR_5-ion_ were separately stained for fibrinogen α, fibrinogen β, C4d, and C4b-BP. There was no observed difference between the non-irradiated and GCR_5-ion_ irradiated mice for any of the markers and there was no significant staining observed in any of the samples.

SUPPLEMENTARY FIGURE S4 Immunohistochemical analysis of type I and type III collagen in mouse myocardium. Myocardial sections from mice, either irradiated with 150 cGy GCR_5-ion_ or age matched non-irradiated controls, were stained for type I **(A)** and type III **(C)** collagen. For each group, n = 2. **(B)** Quantification of the collagen distribution area revealed a more prominent presence of type I collagen in GCR_5-ion_ irradiated mice compared to the control mice. **(D)** However, the type III collagen distribution showed no significant difference between the two groups. All images are at ×40 magnification, with the scale bar indicating 20 μm.
