# Supplementary figures and images for: Proteomic and phosphoproteomic characterization of cardiovascular tissues after long term exposure to simulated space radiation
Source: Front Physiol. 2024 Apr 18;15:1248276. doi: 10.3389/fphys.2024.1248276 (PMC11063234; doi:10.3389/fphys.2024.1248276)

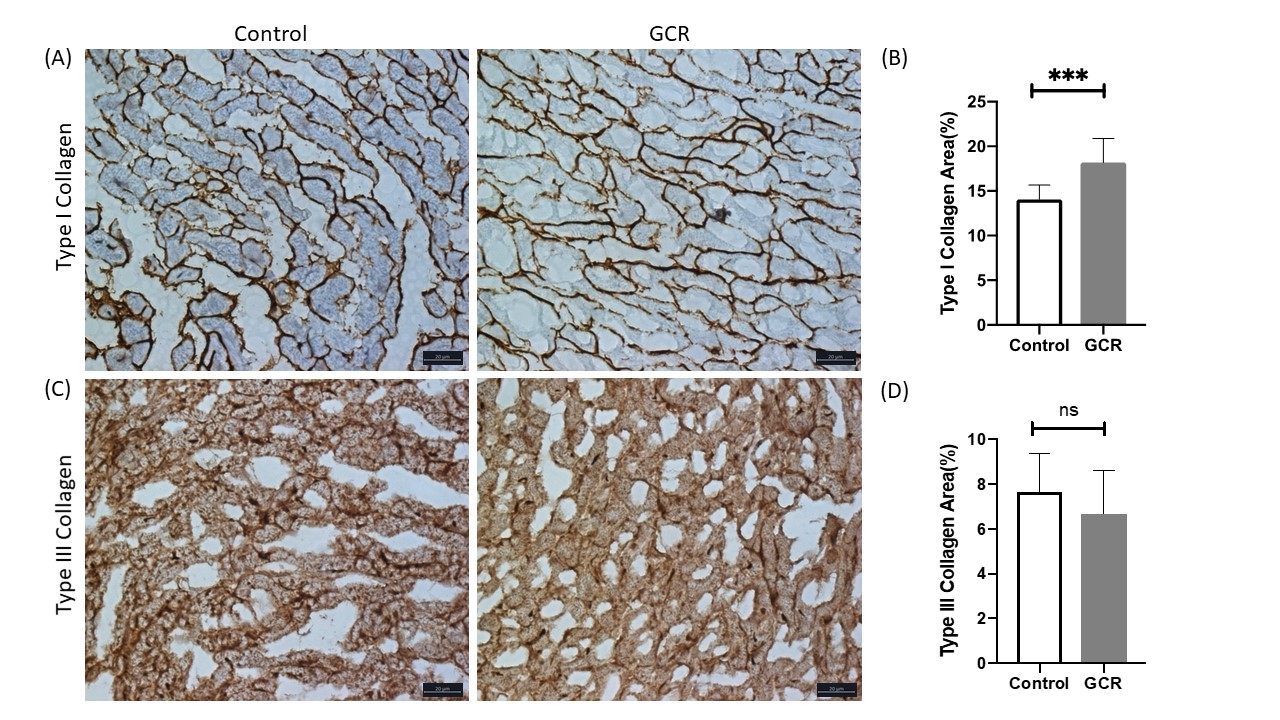

Supplement: Supplementary file 1 [file Image3.jpeg]

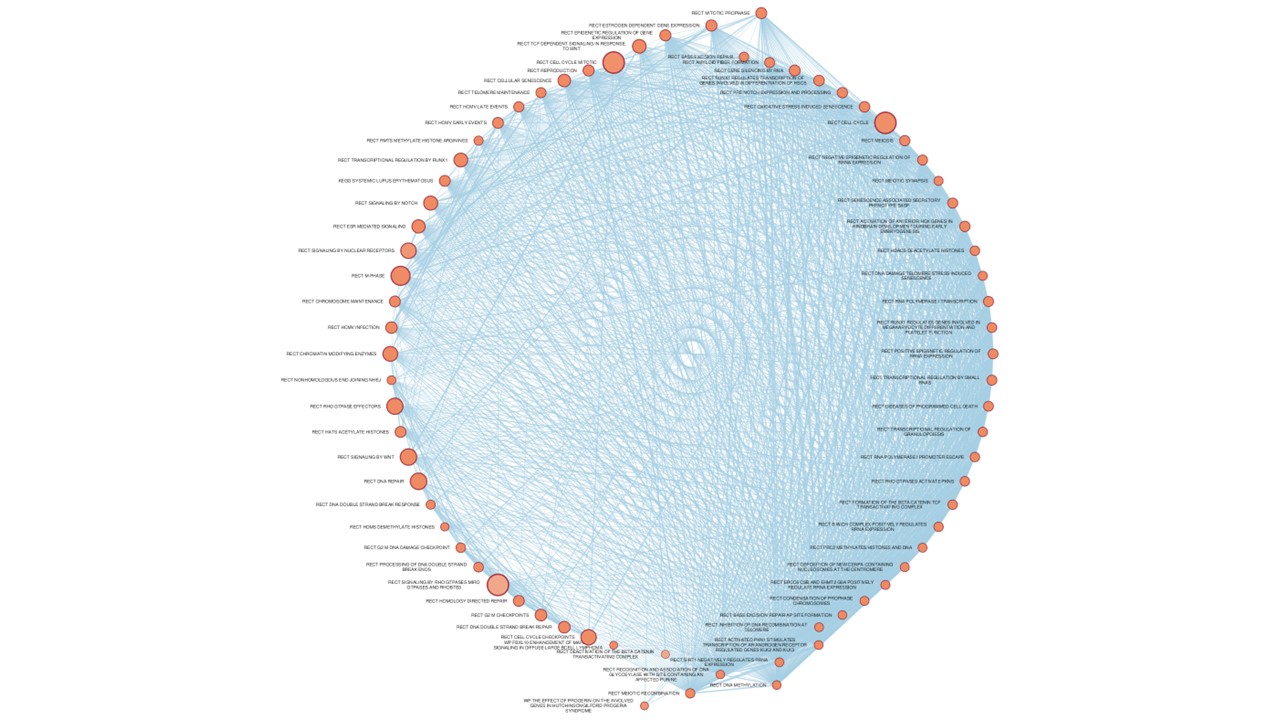

Supplement: Supplementary file 2 [file Image1.jpeg]

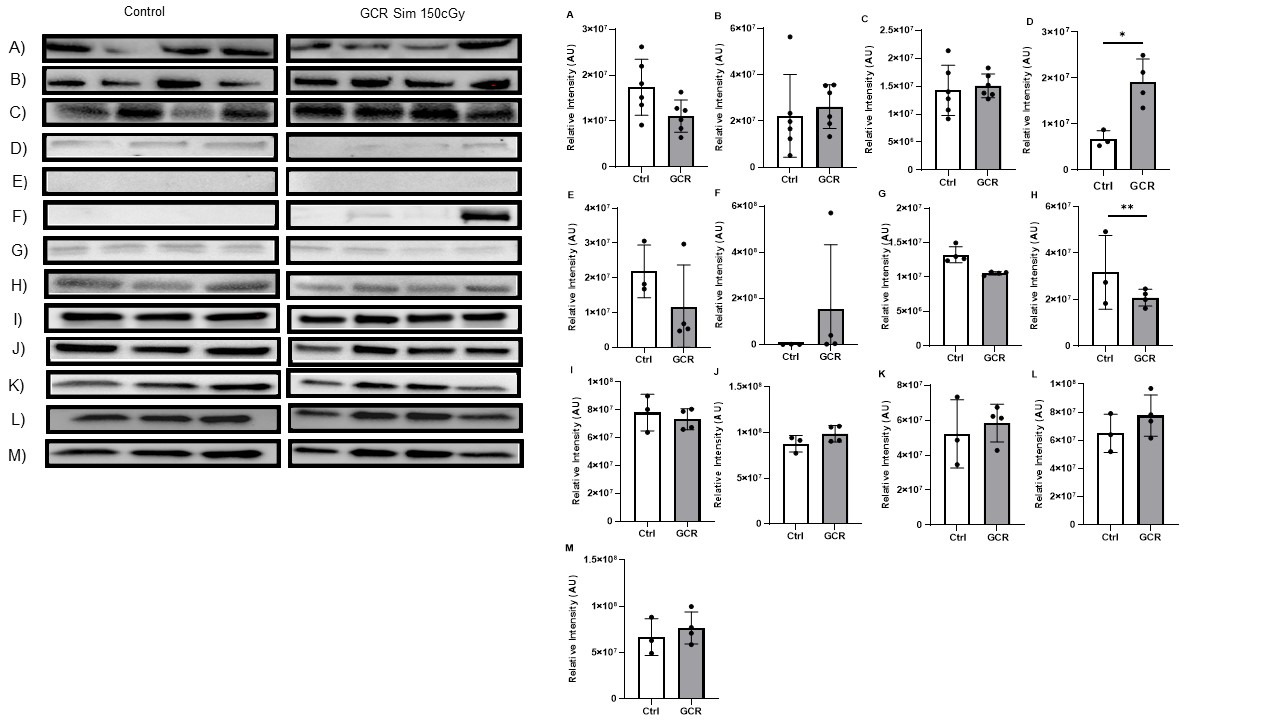

Supplement: Supplementary file 3 [file Image4.jpeg]

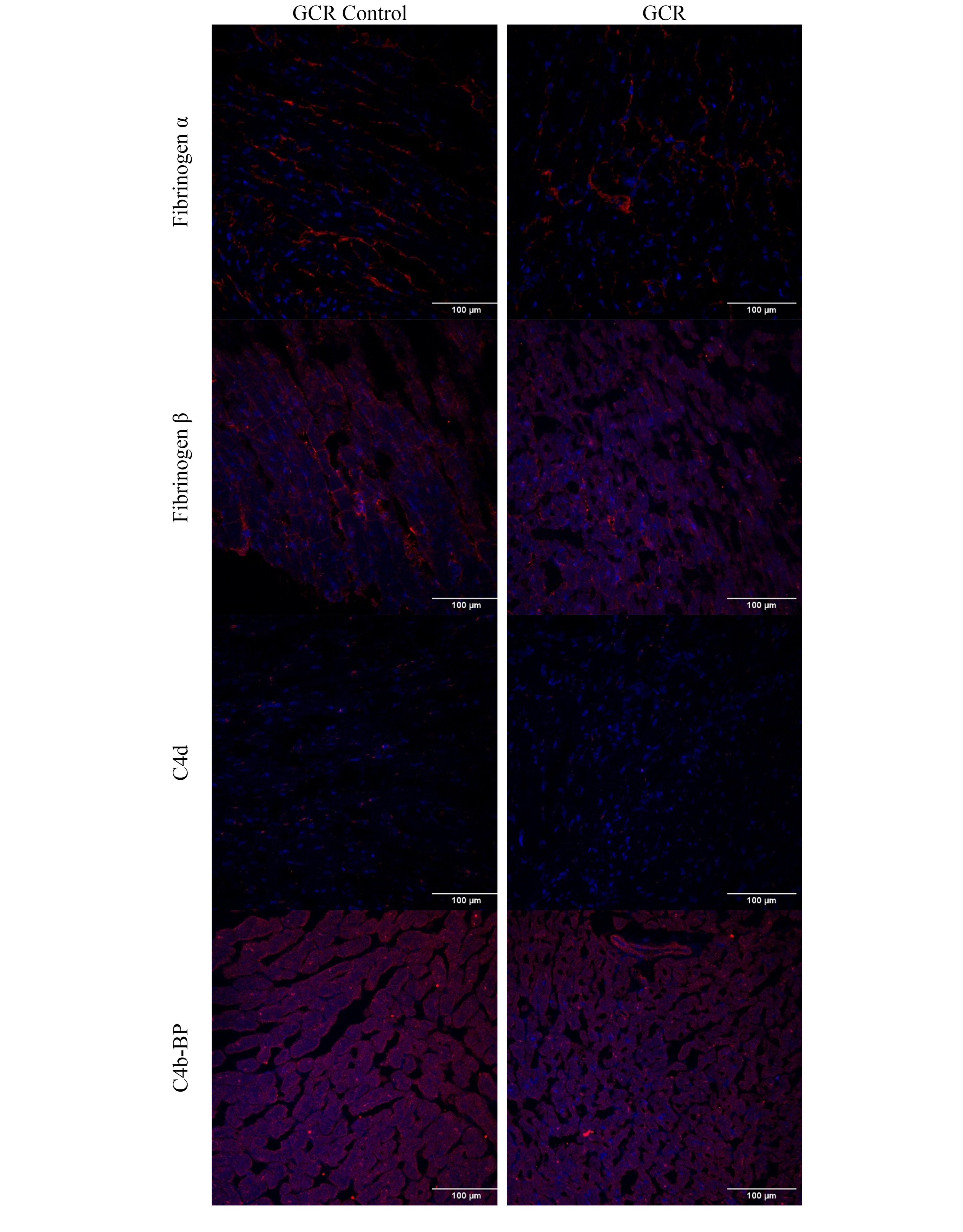

Supplement: Supplementary file 4 [file Image2.jpeg]
